# Flight Performance and Feather Quality: Paying the Price of Overlapping Moult and Breeding in a Tropical Highland Bird

**DOI:** 10.1371/journal.pone.0061106

**Published:** 2013-05-08

**Authors:** Maria Angela Echeverry-Galvis, Michaela Hau

**Affiliations:** 1 Department of Ecology and Evolutionary Biology, Princeton University, Princeton, New Jersey, United States of America; 2 Evolutionary Physiology Group, Max Planck Institute for Ornithology, Am Obstberg 1, Radolfzell, Germany; 3 Department of Biology, University of Konstanz, Konstanz, Germany; University of Milan, Italy

## Abstract

A temporal separation of energetically costly life history events like reproduction and maintenance of the integumentary system is thought to be promoted by selection to avoid trade-offs and maximize fitness. It has therefore remained somewhat of a paradox that certain vertebrate species can undergo both events simultaneously. Identifying potential costs of overlapping two demanding life history stages will further our understanding of the selection pressures that shape the temporal regulation of life history events in vertebrates. We studied free-living tropical Slaty brush-finches (*Atlapetes schistaceus*), in which individuals spontaneously overlap reproduction and moult or undergo both events in separation. To assess possible costs of such an overlap we quantified feather quality and flight performance of individuals in different states. We determined individual’s life history state by measuring gonad size and scoring moult stage, and collected a newly grown 7^th^ primary wing feather for later analysis of feather quality. Finally, we quantified flight performance for each individual in the wild. Overlapping individuals produced lighter and shorter wing feathers than individuals just moulting, with females decreasing feather quality more strongly during the overlap than males. Moreover, overlapping individuals had a reduced flight speed during escape flights, while their foraging flight speed was unaffected. Despite overlappers being larger and having a smaller wing area, their lower body mass resulted in a similar wing load as in breeders or moulters. Individuals measured repeatedly in different states also showed significant decreases in feather quality and escape flight speed during the overlap. Reduced escape flight speed may represent a major consequence of the overlap by increasing predation risk. Our data document costs to undergoing two life history stages simultaneously, which likely arise from energetic trade-offs. Impairments in individual quality and performance may represent important factors that select for temporal separation of life history stages in other species.

## Introduction

Most long-lived animals undergo important life history stages such as reproduction, maintenance of the integumentary system in vertebrates, and quiescent periods in a specific sequence each year. A temporal separation of life history stages is assumed to be advantageous because if each stage is energetically costly, its simultaneous expression would compete for crucial resources with another stage expressed at the same time [1]–[8]. However, individuals of some vertebrate species are able to temporally overlap costly life history stages. Some mammal species overlap phases of breeding and pelage change [1], or part of their reproduction with migration [9], [10], while some bird species are able to temporally overlap breeding and moult [11]–[18].

If overlapping two major life history stages is too costly for many species, the question arises as to how individuals that display the overlap are able to cope with this challenge? This issue has been tackled in avian species, in particular tropical birds where individuals from a considerable number of species can display a complete and extended co-occurrence of both events [14]–[18]. Based on this work, it has been proposed that species may be able to afford the overlap when [14]: a.) environmental conditions are favourable – either because of an overabundance of food resources, or because resources that are critical for one of the two life history stages are only limited for a short period of time, b.) compensatory mechanisms are in place, for example if individuals can reduce the intensity of both life history processes when they co-occur to minimize trade-offs. Indeed, many tropical bird species do display both processes at low intensity, i.e. they grow new feathers slowly, lay small clutches, and have extended incubation periods [19], [20]. Also, tropical bird species with long wing moult durations are more likely to display the moult-breeding overlap [18]. Moreover, in captive Zebra finches (*Taeniopygia guttata*) overlapping individuals slow down feather growth rate and moult fewer feathers simultaneously compared to individuals just moulting [21], indicating that even under the benign conditions of captivity with *ad libitum* food availability individuals try to minimize trade-offs by reducing moult intensity. Conversely, temperate zone species that do not reduce moult intensity or speed when overlapping the final parts of their breeding cycle with moult experience trade-offs by facing lowered overwinter survival and reduced reproductive success in the next breeding season [11], [22]. These findings corroborate the hypothesis that the moult/breeding overlap is costly and if a reduction in the intensity of both processes does not occur, major trade-offs will ensue. However, it has not yet been tested whether tropical birds species, which often undergo both reproduction and moult at a slow pace (i.e. at low intensity) are indeed able, under natural conditions to compensate for potential costs arising from an extended and complete moult/breeding overlap. Also the nature of such potential costs has remained unclear– which likely represent the selection factors that promote a separation of both events in other species.

In the present study, we studied Slaty brush finches (*Atlapetes schistaceus*, Boissonneau, 1840), from a wild Neotropical population to determine whether costs would be detectable in overlapping individuals. Individuals in this population can spontaneously either display the overlap, just moult or just breed. Reproductive success is often difficult to quantify in tropical bird species due to unknown or inaccessible nesting sites and large territory sizes [23]. By contrast, a potential cost that can be readily quantified and would have major fitness implications in avian species would be an impairment in feather quality. Moult is energetically costly [24], [25], and feather growth requires substantial protein metabolism. Insufficient protein supply during moult can even result in “fault bars”, i.e. areas of low keratin density that makes feathers weak and prone to breakage [26]. High quality plumage, especially in wing feathers, improves flight speed and manoeuvrability and thereby increases the likelihood to escape predation [27]–[29]. High quality plumage could also decrease the costs of foraging activities, and would to some extent, improve thermal insulation [30], [31]. To our knowledge, possible consequences of the overlap either for moult processes (e.g., feather quality), or a resulting phenotype (e.g., flight performance) have not been quantified in a species, especially in the wild.

If the overlap was costly, we predicted that overlapping free-living Slaty brush-finch individuals would (1) produce feathers of lower quality based on structural measurements, (2) have increased wing load, and (3) show reduced flight performance. We also predicted that (4) costs to either feather quality or flight performance would be more prominent in females, which may face greater demands than males during reproduction due to egg production. To test these predictions we determined the reproductive condition of free-living individuals by direct examination of the gonads, and took detailed records of flight feather moult. We further quantified functional traits like feather quality and wing load, as well as flight performance in individuals showing the overlap versus individuals just in moult or just breeding. We aimed at repeatedly monitoring the same individual in different states, to address the question of whether possible costs of the overlap are due to the overlap or to differences in the quality of individuals.

## Methods

### Study Species and Site

The Slaty brush-finch is a forest-dwelling bird species widely distributed in Andean montane cloud forests above 2200 m, where it forages on insects in the sub-canopy and understory areas. At our study site, Sabana de Bogotá, Colombia (04°36 N; 74°18′ W, 2750 m a.s.l.) only one breeding period has been recorded that lasts from mid-July to early November, during which females lay a single clutch of 2 eggs [23]. A sex dimorphisms has not been found in either size or plumage in this subspecies (A. *schistaceus schistaceus*) [23], including the specific population we studied [32], and MAEG unpublished data].

In 2000, and from 2008–2011, we captured free-living individuals from July to November each year. Upon capture, individuals were weighed and had their tarsus lengths measured. Individuals were classified as either adult or juvenile based on skull ossification, and only individuals with fully-ossified skulls (i.e. adults) were included in our analyses. All individuals were ringed with a unique combination of a numbered band and darvic colour bands before being released again. A total of 132 adults were caught during our study. However, not all of them could be included in the analyses (see Statistical Analyses for details).

The wing feather tract of Slaty brush-finches has nine primary feathers and 9 secondary feathers (of which three of the internal feathers are considered to be tertials). Wing and tail feathers were individually examined for moult and scored from 0 (missing) to 5 (complete new feather); a slight modification from British Trust for Ornithology (BTO) to evaluate moult intensity [33]. Based on this score, a moult intensity index was computed (Córdoba-Córdoba & Echeverry-Galvis, *unpublished data*.), which relates the total moult value of wing feathers and tail feathers to the total number of flight feathers, as well as to a complete post-moult value (moult intensity index =  [(Σ wing feather scores/number of feathers)/Σ tail feather scores/number of feathers)]-10). This index ranges from 0.0 to 0.50, where individuals with a value greater than 0.02, and symmetrical feather replacement, were considered to be actively moulting. The threshold value of 0.02 in this index refers to individuals either at the start or at the end of moult based on the BTO coding and analysis method (see Figure S1). The number of primary feathers growing simultaneously (in any state of growth) was recorded as well for each individual.

Reproductive state was determined by measuring gonad size via unilateral laparotomy under a light Isoflurane anaesthesia [34]. Length and width of the left testis was measured with calipers to the nearest 0.1 mm and its volume was calculated using the ellipsoid-cylinder formula; for females, the diameter of the biggest follicle was measured to the nearest 0.1 mm. Testis sizes showed a bimodal distribution, with volumes in one group ranging from 1.00–9.10 mm^3^ (assigned as “non-breeders”), and in another group from 141.11–201.06 mm^3^ (assigned as “breeders”). For females, a similar bimodal distribution was found, with non-breeding females having follicle diameters between 0.5–2 mm, and breeding females having one follicle of at least 7 mm in diameter. Individuals with enlarged gonads and active moult were termed “overlappers” (and will be called so throughout the paper), birds displaying enlarged gonads but no moult were termed “breeders”, and birds with regressed gonads but active moult were termed “moulters”. We did not detect a sex bias in individuals assigned to the different states (Chi-square: p≥0.3 for each state).

### Feathers and Wing Load

For all captured individuals during the field periods of 2008–2011, regardless of their state, the 7th primary (p7) of the left wing was plucked if it was a newly moulted and fully grown, intact feather. Each feather was weighed to the nearest 0.001 g (Damal DIA-50 scale) immediately after being plucked, and then stored individually in a glassine envelope. Individual feathers were subsequently scanned using a high-resolution Epson-Expression 10000XL scanner and the image was analysed using ImageJ software (http://rsbweb.nih.gov/ij/). From these images we determined (1) rachis length (mm), and (2) total feather area (mm^2^, with both vexiles and rachis included).

For individuals captured in 2010 and 2011 wing-load was estimated as body mass/wing area. For this, the extended right wing of each individual was digitally photographed and later measured using ImageJ software; total wing area was then calculated following [35]. Repeatability of this measurement [36] was determined using three photographs made per individual. Wing area was significantly repeatable (r = 0.40, F = 4.45, df = 32, p = 0.002).

### Flight Performance

Behavioural observations were conducted whenever a free-living, individually marked individual was encountered during a bout of fieldwork (which lasted three to four continuous days). Only individuals for whom state was determined within three days of their flight performance measurements were included in the analyses. Since all birds for which flight speed was determined were already individually marked, we were able to assess the performance of some individuals in a blind way (thus avoiding any possible observer bias). Observations took place between 5∶00 to 19∶30 and lasted on average 10 minutes (range 3–17 minutes). Flight speed was determined by measuring the distance from the initial departure point to the first contact point that the bird made with any vegetation in a straight-line flight, using a laser range finder (Leica D3). A precision stopwatch (Casio HS-80TW) was used to determine the time travelled between the two points (similar to [62]). The speed of two types of flight was evaluated during these observations. “Normal flight” was recorded when in the course of the behavioural observations an individual moved within the understory by actively flying (no hopping or jumping) without being chased or scared. If multiple normal flight speeds were recorded for one individual within one observation period (ranging from 1–6 normal flights per observation bout), the data were subsequently averaged. The second type of flight, “escape flight”, was recorded either at the time of release after capture; or when an individual was actively chased or scared by loud noises purposely made by observers. No significant difference was found between the speed of escape flight after release and during a chase (t-test t_67_ =  −0.99 p>0.3), therefore they were lumped. A total of two observers were involved in the flight speed measurements. No difference between observers could be detected in the speed of escape flight at release time (t-test t_30_ =  −0.17 p>0.8) or in normal flight (Wilcoxon rank-sum: S = 73, p = 0.9), so data from both observers were lumped.

All animal protocols were approved by the Institutional Animal Care and Use Committee of Princeton University, protocol 170, and under the National Permit 002 of 2008 CAR to MAEG.

### Statistical Analyses

Six individuals were found to be in a quiescent state (neither in moult nor in breeding condition), and were excluded from further analyses due to small sample size. A total of 13 individuals were captured repeatedly in different years (ranging from one to four recaptures), a fact that we accounted for in our statistical analyses (see below), but also utilised to analyse whether costs were detectable on an individual level.

### Morphology and Body Characteristics

Morphological measures were compared between individuals of different states using an ANCOVA with body mass (as a proxy for body condition) as a covariate, followed by pair-wise comparison Tukey-Kramer-HSD post-hoc tests. For individuals recaptured in the same state, a mean value was computed and used in these analyses. Although there is still an on-going debate as to the most appropriate proxy for body condition [37]–[39], body mass has been proposed to be a better proxy than other indices [40].

### Feathers and Wing Load

Even though we plugged a newly moulted p7 from all individuals after capture, data from breeders were not included in the statistical analyses of feather quality analyses since it was not possible to determine the age of their flight feathers. Thus, a total of 35 moulters and 36 overlappers were included in these analyses.

For statistical analyses, feather mass was normalised for feather size in two ways: first by dividing it by raquis length (or calamus length), and secondly by dividing it by the total vane area. These normalized values were then used as proxies for feather quality in subsequent analyses (see also [41], [42]). ANCOVAs with body mass and sex as covariates were used to test for differences in the two normalised feather mass variables among individuals of different states.

There was no sex difference in wing load for each states (p≥0.24 for all states), so sexes were pooled and wing load was compared between states using an ANOVA.

### Flight Performance

We used separate Generalized Linear Mixed Models (GLMMs) for the two types of flight to explore which factors explained individual differences in flight speed. In these models we included the variables: state, feather mass, sex, body mass, tarsus length, wing load, year, distance travelled, and the interactions state*sex and year*state as variables with fixed effects. Individual ID was included a random factor, since some individuals were assessed more than once for a given flight type. Variables with non-significant effects in the model (p>0.1) were removed in a step-wise manner to generate the most parsimonious final model (shown in Table 1).Post-hoc pair-wise Tukey-Kramer HSD tests were performed when the overall model revealed significant effects. For escape flights, we had a total of 69 observations (21 observations for moulters, 22 for overlappers and 26 for breeders) with no sex difference in the number of observations per state (Chi-square: p≥0.4). For normal flights, we had 71 observations (27 for moulters, 23 for overlappers and 21 for breeders). We did detect a sex difference in all states (Chi-square: p≤0.046 for each state), with more females observations for overlappers and moulter and for breeders, in which male observations were more. All statistics were run in JMP 10.0 and significance was accepted at p≤0.05. All data are presented as mean ±1SEM.

**Table 1:** Variables included in the final GLMM model analysing the speed of: a) escape flight and b) normal flight.

Dependent variable	Explanatoryterms	Adj. R^2^	Estimate	SE	df	F	p
a) Escape flight		0.69					
	State∞		0.83	0.02	2	10.63	0.005
	State		0.46			12.63	0.002
	Feather mass		0.45	0.02	1	6.54	0.01
	Sex		−1.41	0.23	1	1.9	0.06
b) Normal flight		0.48					
	State∞		0.78	0.53	2	0.87	0.59
	State		0.61			0.58	0.60
	Feather mass		0.70	0.75	1	5.29	0.042

∞State: whether an individual was a moulter, a breeder or an overlapper; for post-hoc test please see text. For details on all variables included into each statistical model please see Statistical Analysis in text; variables with p>0.1 were not included in the final model.

## Results

### Morphology

Individuals of different state differed in their tarsus length (F_2,104_ = 14.94, p<0.002), with overlapping individuals having longer tarsi (29.1±0.1 mm) than individuals of any other state (breeders 28.5±0.1 mm, moulters 28.2±0.1 mm; Tukey-Kramer HSD: overlappers-breeders: p = 0.003, overlappers-moulters: p<0.0001). Body mass also differed with state (F_3,57_ = 7.59, p = 0.0008); despite being structurally larger (having longer tarsi), overlappers were lighter for their size (28.3±0.1 g) than both moulters (30.1±0.2 g) and breeders (30.7±0.3 g) (Tukey-Kramer HSD: p = 0.008; p<0.0005; respectively).

### Feather Quality and Wing Load

A detailed description of the moult sequence in this species is still lacking. Our data indicate that moult progressed – as expected for oscine passerines – in a standard sequential manner or descendant sequence, starting from the innermost primary (p1) and continuing with a sequential replacement toward the end of the wing (p9). Moulting and overlapping individuals were caught throughout the study period in various stages of their full primary replacement.

**Figure 1:**
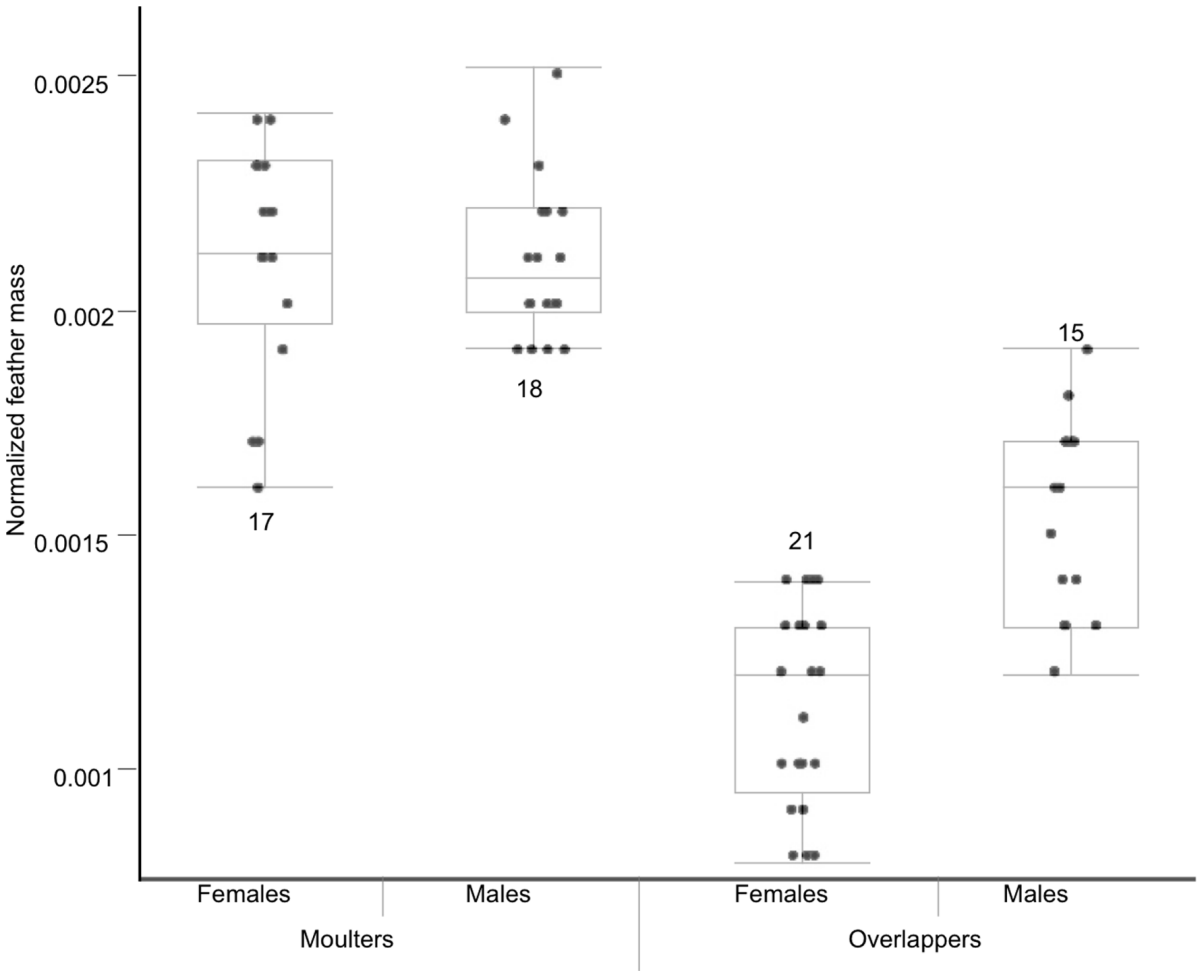
Normalised feather mass (by feather length) of the 7^th^ primary for individuals in different states. Box and whisker plots show median, range, first and third quartiles. Filled circles indicate individual data points. Numbers indicate sample sizes.

Feather mass normalised by feather length differed with state (F_3,70_ = 92.4, p<0.0001), with feathers of overlapping individuals being of almost half the mass of those grown by moulters (Fig. 1). Similar results were obtained when feather mass was normalised by vane area (F_3,70_ = 104.6, p<0.0001). Reduced feather mass (normalised by feather length) was most obvious in overlapping females (0.008±0.00002 g), which had significantly lighter p7 than overlapping males (0.011±0.0002 g; post-hoc Tukey-HSD p = 0.0002). There was no difference in normalised feather mass between the sexes within moulters (post-hoc Tukey-HSD p>0.74). These findings were corroborated by the data from recaptured individuals, in which individuals overlapping in a given year had consistently lighter p7 (0.009±0.0005 g; feather length 7.58±0.1 mm) than in a different year when being a moulter (0.014±0.001 g; 7.75±0.05 mm) (Wilcoxon Signed Rank: S =  −10.5, p = 0.03, n = 10; sexes lumped due to small sample sizes).

Body size (tarsus length) and normalised feather mass did not correlate, either when analysed separately for each state (per state: r≤0.17, p≥0.29) or when all individuals were lumped, irrespective of their state (r = 0.12, p = 0.26). Likewise, body mass did not correlate with normalized feather mass (per state: r≤0.32, p≥0.19, states lumped r<0.58; p>0.36).

**Figure 2:**
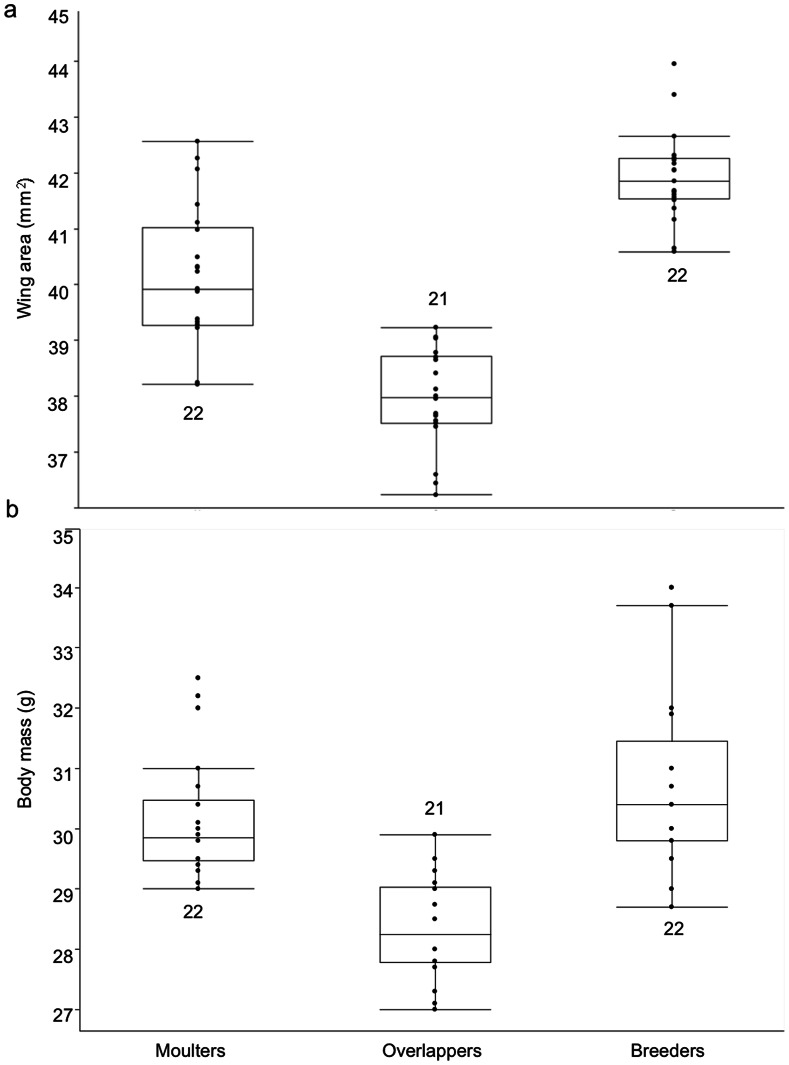
Wing load components. a) wing area (mm^2^) and b) body mass (g) for individuals in different states. Box and whisker plots show median, range, first and third quartiles. Filled circles indicate individual data points. Numbers indicate sample size.

Wing load did not differ according to state (F_3,66_ = 0.86 p = 0.42). However, wing area differed between individuals of different states (F_2,64_ = 53.8, p<0.0001), with breeders having, as expected, larger wing areas than individuals in the two other states that were in active moult (Tukey-Kramer-HSD: p≤0.001). However, inverse differences in body mass with state (as noted previously) resulted in similar wing loads across states (see Morphology Results). Wing area was positively correlated with body mass (r = 0.50, p<0.0001; see also Figs. 2a and b).

### Flight Performance

**Figure 3:**
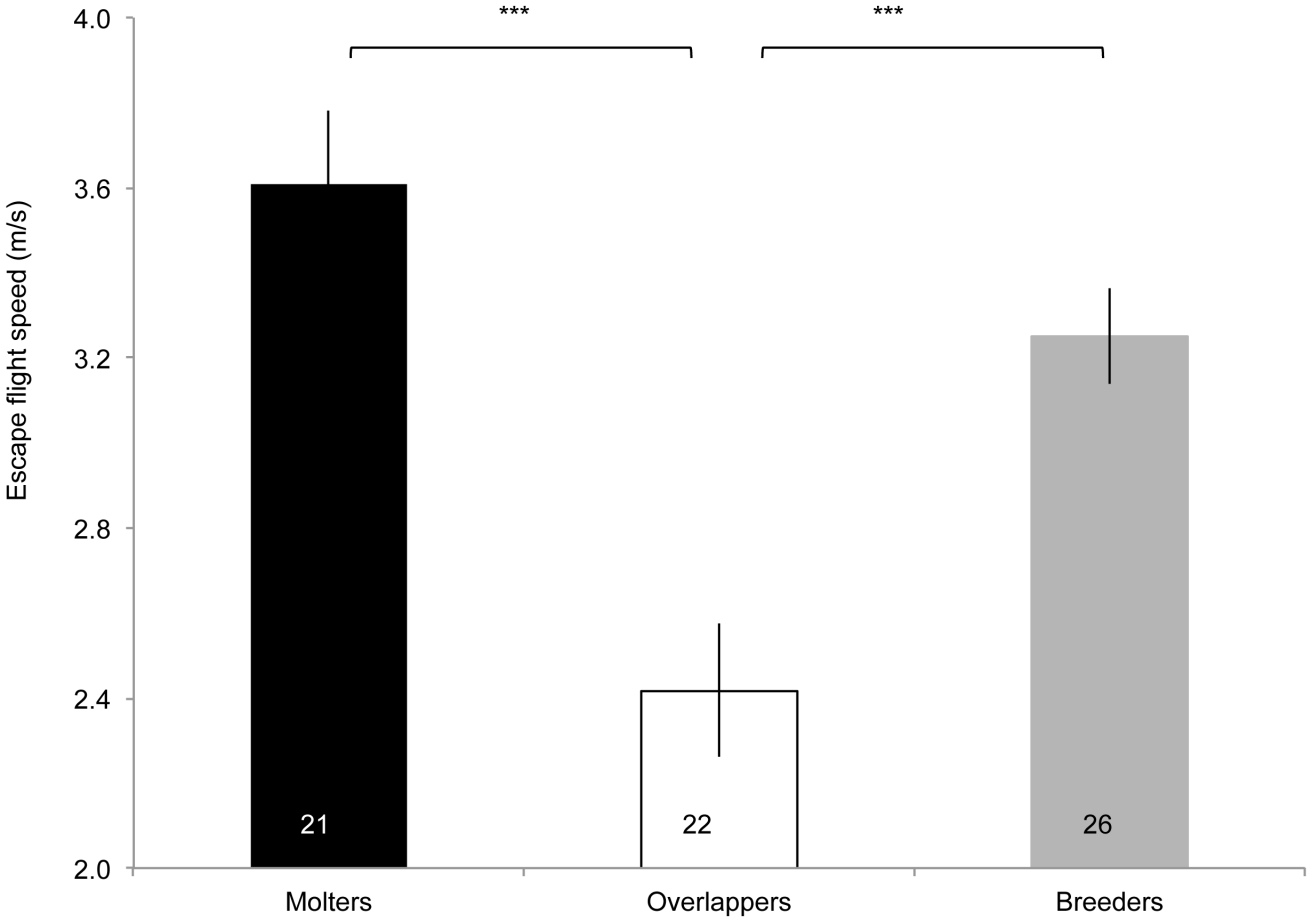
Escape flight speed (m/s, mean ± SE) according to state (***p<0.0001). Numbers inside bars indicate sample sizes.

State was the variable that best explained speed of escape flight (Table 1), with overlapping individuals being about 30% slower during escape flights than moulters or breeders (Tukey-Kramer HSD: overlappers vs. breeders p<0.0001, overlappers vs. moulters p = 0.0002; Fig. 3). Furthermore, individuals carrying feathers with greater mass showed a faster escape speed (Table 1). By contrast, individuals of different states did not differ in their speed of normal flight (Table 1). However, again feather mass significantly explained normal flight speed, with lower feather mass being associated with slower speed during normal flight. There was no difference between moulters and overlappers in the number of feathers in active moult (Chi-square-test: **χ** ^2^_2, 60_ = 0.027, p = 0.8), nor was there a correlation between number of feathers in active moult and the speed of normal flight (Pearson r^2^ = 0.002, p = 0.5).

Among individuals recaptured in different years, a change in state from moulter to overlapper or vice versa was recorded in 7 individuals, while 6 individuals switched from breeding to overlapping or vice versa in subsequent years. Lumping sexes to increase the sample size for recaptured individuals confirmed the patterns described above. Whenever an individual showed the overlap in a given year, its escape speed was reduced by 28–36% compared to a year in which it only moulted or bred (Friedman test, χ^2^_13_ = 11, p = 0.004, n = 15), with no difference for normal flight speed (Friedman test, χ^2^_15_ = 1.2, p = 0.5, n = 17).

## Discussion

The current study demonstrates pronounced impairments in phenotype and performance in individuals of a free-living tropical bird that overlapped reproduction and moult compared to individuals that underwent only one life history stage at a time. During the overlap, wild Slaty brush-finches grew flight feathers of lower mass, an adequate proxy for feather quality [43], [44], and had a slower speed of escape flight than when only moulting. This result was further confirmed by comparing individuals across different years, showing that this effect was not due to differences in individual quality but rather due to trade-offs arising from the overlap. Reduced speed of escape flight may represent a severe fitness cost because it likely increases predation risk [45]–[47]. Our data therefore suggest that the fitness consequences of low feather quality through deficits in flight performance may represent one selective factor that promotes a sequential expression of life history stages in other species.

During the same study period and in the same study area about 80% of the avifauna that we monitored as part of a concurrent study (n = 53 species) also displayed the moult/breeding overlap, suggesting that individuals from a large number of species could be facing similar costs (Echeverry, *unpubl. data*). Other taxa may also incur costs to phenotype and performance when overlapping two life history stages, thus explaining why the strategy of temporal separation of life history stages is so prevalent among vertebrates [2]. The possible severity of the costs identified in the current study raises the question as to why individual Slaty brush-finches would follow a strategy of moult/breeding overlap. We were not able to determine reproductive success, but consider it likely that the benefits of being able to reproduce at the same time as moulting may counter these costs, at least under certain environmental circumstances (see also [19]). Alternatively, though not mutually exclusively, only individuals in a better condition (due to genetic, developmental or ecological circumstances) may be able to bear these costs, as overlapping individuals were generally larger than those only in moult or in a reproductive state.

To date, most of the information regarding flight performance and its dependence on feather quality comes from captive experiments [48], [49] that might underestimate the possible demands and consequences of moult for wild individuals [31], [35], [50]. To our knowledge, the current study is the first one in the wild that directly relates feather quality to flight performance. By adapting behavioural approaches commonly used in other contexts to quantify performance [51]–[54], we were able to compare flight performance among free-living individuals that follow divergent life-history strategies. We should note, however, that other factors not included in our analyses may also affect flight performance in the wild. Although our moult intensity index allowed us to classify individuals into moulters or non-moulters, the specific feather currently moulted or the exact moult stage (whether being at the beginning of the end of moult), might also play a role in the flight performance [55].

Feathers of high quality are important in all stages of a bird’s life [24], [56], [57]. Moult typically leads to improved flight performance [58], while flying with feather gaps in the wings or with severely worn feathers increases energy expenditure [28], [59], [60]. Our data demonstrate that even newly moulted feathers, when produced during the moult/breeding overlap, can lead to reduced flight performance in an escape situation. By contrast, our findings suggest that overlapping Slaty brush-finches were able to compensate for reduced feather quality during normal flight. One possible compensatory mechanism could be the body mass reduction that we observed in overlapping individuals (which had a 9% lower body mass than breeders), which despite their larger size (longer tarsi) and reduced wing area resulted in a similar wing load as in moulters or breeders. Such adjustments in body mass during periods of reduced wing area are known to occur in other bird species as well, for example in tree sparrows (*Passer montanus*) [61] and great tits (*Parus major*) [62]. However, body mass reduction in overlapping Slaty brush-finches appeared to be an insufficient compensatory mechanism during high-powered escape flights. Take-off speed is strongly dependent on the condition of the wings, and individuals with “holey” wings typically have slower take-off speeds [63]. Unlike other studies [58]–[60], [64], we found no difference in body mass between breeders and moulters.

In our study population, as expected in the absence of fault bars in feathers [65], feather mass was correlated with rachis width and length, two traits that relate to bending stiffness and/or resistance to wear [43], [66], [67]. In captive European starlings (*Sturnus vulgaris*) it has been shown that lighter feathers with narrower raches are less rigid and resistant [43], features that are important in determining the aerodynamic properties of wings [68]. These findings point to a mechanistic link, such that lighter and shorter feathers result in aerodynamical impairments that decrease flight speed and possibly survival in overlapping wild Slaty brush-finches. Feather mass is also reduced in birds that have experienced food reduction [69], chronic stress combined with food restriction [26], or long-term administration of the metabolic hormone corticosterone [70]. Hence, energetic trade-offs between moult and reproduction could be a major proximate reason for the impaired feather quality in overlapping individuals in our study. However, it is important to note that the observed correlation between feather mass, rachis width and stiffness/resistance of a feather does not appear to apply to all bird populations. For example, in a migratory population of blackcaps (*Sylvia atricapilla*), where selection is expected to promote feather stiffness, individuals had feathers with thinner raques but a greater resistance to bending than sedentary populations [71]. An improved understanding of the relationship between feather characteristics, their physical properties and aerodynamical performance will require further laboratory and field studies on a diverse array of species.

We had predicted that females would show stronger evidence for costs of the overlap than males [60], since they may face greater energy expenditure during reproduction due to egg production [72], [73]. Females did indeed show a stronger reduction in both feathers mass and length during the overlap compared to males, and there was a trend for females to have slower escape flight speeds (Table 1). The lack of a significant sex difference in flight performance of overlappers could be due to different factors including reproduction in female Slaty brush-finches being less energetically demanding than expected due to the small clutch size, or the ability of females to further compensate in performance for poor feather quality. However, it is also possible that sex differences would be more pronounced during the pre-laying period, a reproductive stage that we could not specifically target in our study. An influence of a female’s specific reproductive stage on flight performance is suggested by the finding that females with larger follicles had slower escape flight speeds (r =  −0.24, p = 0.04), while testis size was not related to male escape flight speed (p>0.4).

Overlapping individuals were structurally larger than conspecifics that separated moult and breeding in time. Structural size could indicate an individual’s quality, which is partly genetically determined, while also reflecting conditions for growth during the nestling period [74], [75]. Larger individuals might also be better foragers, and due to a lower mass-specific metabolic rate may in general be less energetically challenged than smaller conspecifics. Larger individuals may therefore better able to defray the costs of the overlap, at least under certain environmental conditions. Finally, larger individuals may also be less subject to predation, either because of their size, social status or habitat quality, thus experiencing an overall smaller risk to survival. However, it is important to note that even though individuals with bigger tarsi were most likely to display the overlap, repeated assessments of individuals confirmed that feather quality and flight performance were reduced whenever an individual displayed the overlap compared to times when the same individual underwent only one life history stage. This result strongly indicates that the costs of the overlap that we observed in feather quality and flight performance were due to the overlap and did not merely arise from differences in individual quality.

Additional studies are required to improve our understanding of the reasons why certain species, and certain individuals within a species, can display a strategy of moult/breeding overlap, when others undergo moult and reproduction in temporal separation [30], [76]. It is conceivable that decreased feather quality and slower escape flight speed bear less of a risk to survival for species in which the rate of adult predation is low [77]. Furthermore, it is possible that species for which flight performance is not as important for many aspects of their life (e.g. to habitat choice, foraging mode, sedentariness, etc.) are more likely to show this strategy. Curiously, some swift species that rely strongly on flight for all activities nevertheless moult during incubating (C. Collins, *pers. comm*). Clearly, further work on species that show the overlap in the wild is required for better insight into the evolution of the temporal organisation of life history cycles in vertebrates.

## Supporting Information

Figure S1 Moult intensity index versus British Trust for Ornithology (BTO) moult score. In the moult intensity index used in this study, individuals with an index of lower than 0.02 were assigned as ‘moulters’. This value of 0.02 corresponds to values in the ranges of 0–10 and 86–90 in the BTO score, representing individuals that could be either starting or ending their moult.
(TIF)

Text S1 Moult intensity index and moult indexes. Comments regarding moult intensity index used in relation to others proposed and their aim.
(DOCX)
