# Supplementary figures and images for: Flight Performance and Feather Quality: Paying the Price of Overlapping Moult and Breeding in a Tropical Highland Bird
Source: PLoS One. 2013 May 8;8(5):e61106. doi: 10.1371/journal.pone.0061106 (PMC3648541; doi:10.1371/journal.pone.0061106)

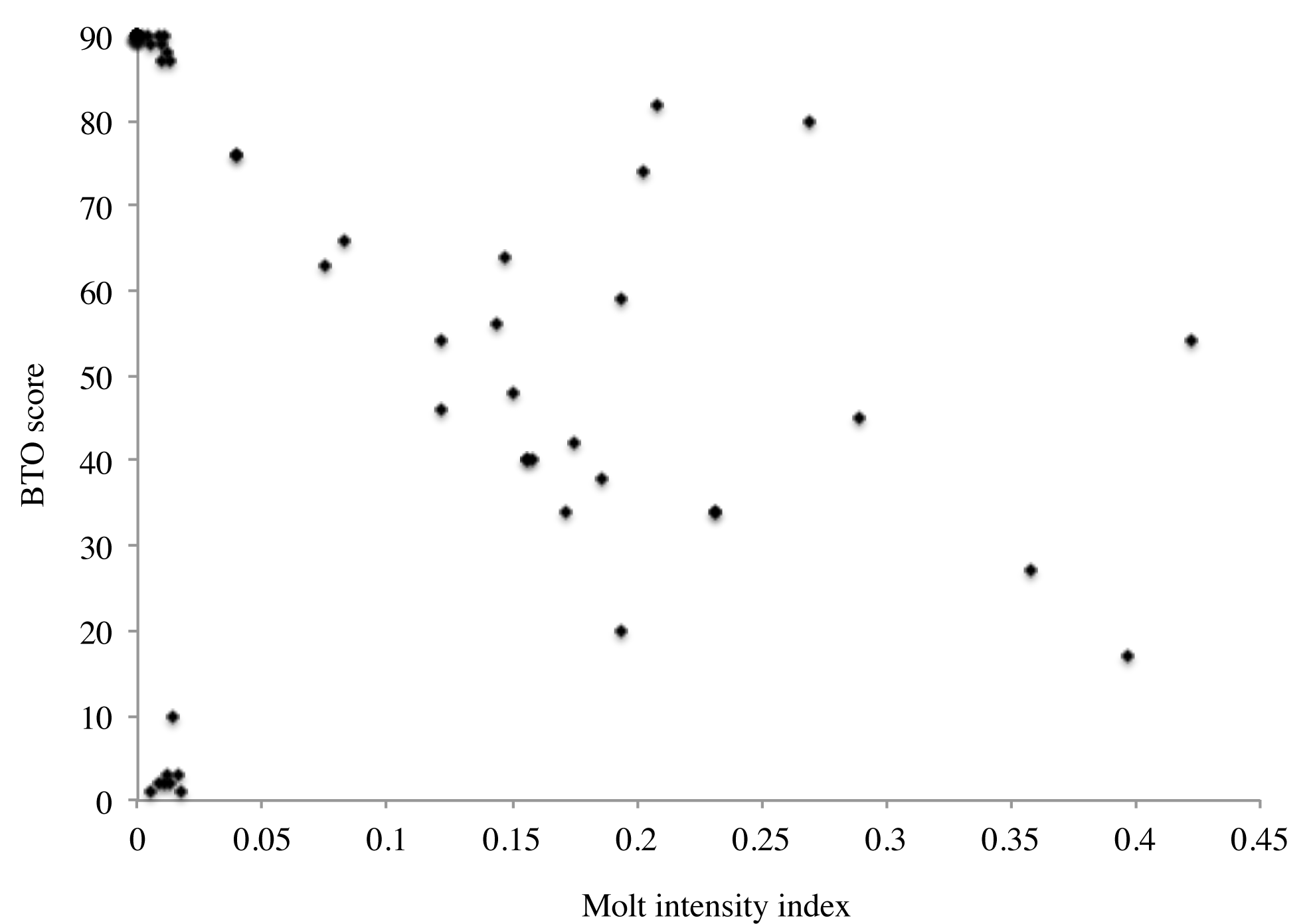

Supplement: Figure S1 — Moult intensity index versus British Trust for Ornithology (BTO) moult score. In the moult intensity index used in this study, individuals with an index of lower than 0.02 were assigned as ‘moulters’. This value of 0.02 corresponds to values in the ranges of 0–10 and 86–90 in the BTO score, representing individuals that could be either starting or ending their moult. (TIF) [file pone.0061106.s001.tif]
