# Supplementary material for: Flight Performance and Feather Quality: Paying the Price of Overlapping Moult and Breeding in a Tropical Highland Bird
Source: PLoS One. 2013 May 8;8(5):e61106. doi: 10.1371/journal.pone.0061106 (PMC3648541; doi:10.1371/journal.pone.0061106)
Supplement: Text S1 — Moult intensity index and moult indexes. Comments regarding moult intensity index used in relation to others proposed and their aim. (DOCX) [file pone.0061106.s002.docx]

Supporting Information

The moult intensity index used in this study closely resembles the raggedness method proposed by Bensch and Grahn [1], which bases the estimation of moult progression on the wing area missing and the speed of feather growth. We were not able to use the raggedness index in the current study, because it requires a priori information on moult details (such as individual feather growth rate, which would further be corrected by feather mass according to other estimates).

The moult intensity index used in our study does not aim at evaluating moult duration or any other moult parameter. The index instead estimates the moult intensity from data on moult of both the wings and the tail. Most other moult indexes that estimate the progression and/or the duration of the process

[1-4] focus exclusively on the wing.

The moult sequence and patterns of many Neotropical species are not fully understood or even unknown, including details like whether they show arrested moult, moult once or twice per year [5,6]. Although it is possible that the moulting patterns of some species closely resemble those of related species for which such information is available, such assumptions could obscure interesting deviations from common patterns. Our moult intensity index aims at assigning individuals to being in moult or not in moult by using an intensity value that: 1) does not require knowledge of the moult sequence, 2) does not incorporate the speed of feather replacement, so no prior information regarding the feather growth rate is needed, 3) takes into account other feather tracts (secondaries, tertials and tail) to provide a holistic view of the flight feather moult for an individuals at a given time.

1. Bensch S, Grahn M (1993) A new method for estimating individual speed of molt. The Condor 95: 305–315.

2. Ginn HB, Melville DS (1995) Moult in birds. 19(null) ed. London: British Trust for Ornithology.

3. Dawson A, Newton I (2004) Use and validation of a molt score index corrected for primary feather mass. Auk 121: 372–379.

4. Underhill LG, Zucchini W (1988) A model for avian primary moult. Ibis 130: 358–372.

5. Wolfe JD, Chandler R, King D (2009) Molt patterns, age, and sex criteria for selected highland Costa Rican resident landbirds. Ornitol Neotrop 20: 451–459.

6. Ryder TB, Wolfe JD (2009) The current state of knowledge on molt and plumage sequences in selected neotropical bird families: A review. Ornitol Neotrop 20: 1–18.
